# Identifying keystone taxa and metabolisms of epilithic biofilms is crucial to the conservation of stone heritage from biodeterioration

**DOI:** 10.3389/fmicb.2025.1600865

**Published:** 2025-05-27

**Authors:** Chenchen Ma, Xiaoying Zhang, Fasi Wu, Xiaobo Liu

**Affiliations:** ^1^School of Environmental and Biological Engineering, Nanjing University of Science and Technology, Nanjing, China; ^2^Department of Conservation Research, National Research Center for Conservation of Ancient Wall Paintings and Earthen Sites, Dunhuang Academy, Dunhuang, China

**Keywords:** biodeterioration, epilithic biofilms, stone heritage, keystone taxa, heritage conservation

## Abstract

**Background:**

Outdoor stone heritage accounts for a large portion of UNESCO World Heritage Sites and is an essential carrier of the ancient civilization of our society. Unfortunately, they usually suffer from serious biodeterioration by diverse microbial colonizers, especially when the environment is available. As microbial communities evolve with the environment, it is important to link the bio-deteriogens to biodeterioration processes accurately.

**Methods:**

We used an integrative high-throughput sequencing and comparative metabolomic approach to unravel the biodeterioration of the Leizhou Stone Dog monuments.

**Results:**

The divergence and similarity of the composition of microbial biofilms colonizing the monuments indicated that photoautotrophic bacteria (e.g., *Leptolyngbya*, *Chroococcidiopsis*, and *Chloroplast*) and nitrifying archaea (e.g., the family *Nitrososphaeraceae*) and/or bacteria (e.g., *Massilia* and *Bacillus*) are the keystone taxa governing the biodeterioration processes. Further, the correlation between the keystone taxa and physicochemical properties confirmed the consistency of the observations of the keystone metabolisms involved in the biodeterioration processes.

**Conclusion:**

Our study highlights the necessity of a case-by-case diagnosis of the keystone taxa and metabolisms before any therapy, advancing the conservation science of cultural heritage.

## 1 Introduction

As early as prehistoric times, rocks and carved stones have been used for making daily tools, designing totems, and building shelters worldwide. For this reason, stone monuments are considered the most crucial evidence of the past civilization of our society, as indexed by the UNESCO World Heritage List.^1^ Unfortunately, most stone monuments have been exposed to an outdoor environment, leaving them at long-term risk from frequent environmental fluctuation, increasing atmospheric pollution, and climate change. Further, the changing environment has dramatically exacerbated both abiotic and biotic deterioration processes of outdoor stone monuments. Notably, geomicrobiological activity and impact often evolve with changing environmental factors, increasing the difficulty of conserving stone monuments.

In most cases, outdoor stone monuments are substantially colonized by colorful biofilms (Villa et al., 2022). The epilithic biofilms are composed of archaea, bacteria, fungi, lichens, and microalgae with different metabolic functions and trophic manners (Liu et al., 2020; Wu et al., 2021). For example, phototrophic microorganisms, usually predominated by cyanobacteria, have been widely observed to grow on stone monuments (Rossi et al., 2012; Zhang et al., 2024). These microorganisms can grow on outdoor stone monuments only relying on the availability of water (Wang et al., 2025), and they are commonly reported as the pioneers of microbial colonizers (Bellinzoni et al., 2003). Together with phototrophs, some chemolithotrophs (e.g., ammonia-oxidizing bacteria or archaea) can convert CO_2_ into organic carbons to support the growth of subsequent heterotrophs (e.g., most bacteria and fungi) in the biofilms. Collectively, the members inside the biofilms drive the geochemical cycles of carbon, nitrogen, and sulfur that are mainly from atmospheric deposition (Mitchell and Gu, 2000), resulting in the continual deterioration of stone by biogenic acids (Liu et al., 2020; Qian et al., 2024). For example, our previous work showed that ammonia-oxidizing archaea and bacteria cooperatively contribute to the production of nitric acid responsible for the biodeterioration of stone monuments at the Bayon Temple of Angkor Thom in Cambodia (Meng et al., 2016). We also found that sulfur-oxidizing bacteria (e.g., *Sulfurovum* and *Acidiphilium*) release sulfuric acid, resulting in the blackening of basalt sculptures of the Leizhou Stone Dog monuments (Wang and Liu, 2021).

Thanks to the multi-omics techniques, we can obtain much microbial information on deteriorated monuments using minimal samples. Although these culture-independent approaches allow us to rapidly construct a public database of microbial information on the biodeterioration of stone monuments, we need a case-by-case analysis of the monuments as they are exposed to different environments. However, we proposed the research priority of the keystone taxa in the biofilms, which act as drivers of microbiome structure and functioning (Banerjee et al., 2018) and as good indicators of pending community shifts (Herren and McMahon, 2018).

**Figure 1:**
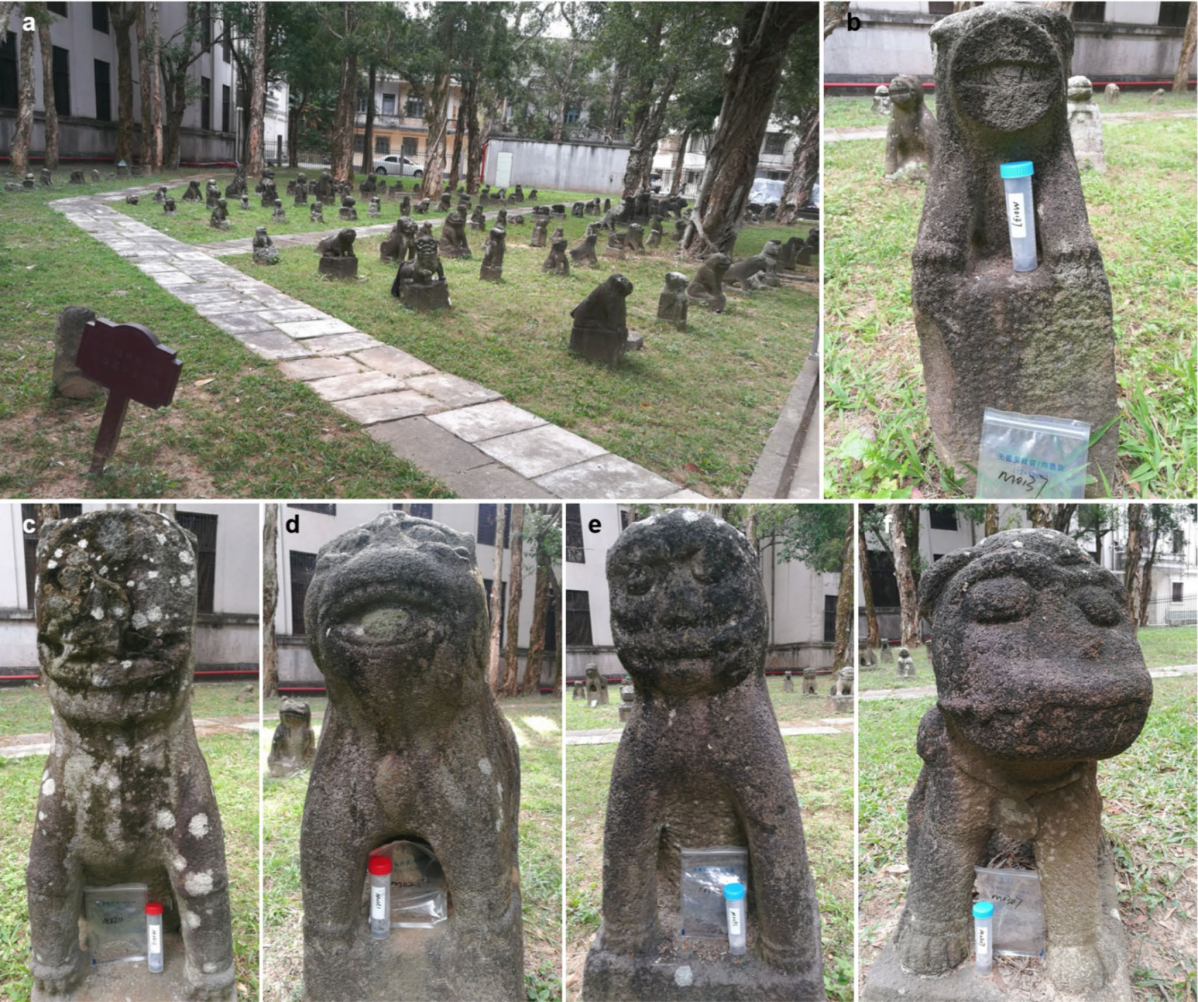
Sculptures of the Leizhou Stone Dog. **(a)** An overview of the sculptures displayed in a courtyard; **(b–f)** are the appearances of the sculptures M0137, M0211, M0251, M0295, and M0307, respectively.

Our focus is on the Stone Dog monuments located at the Leizhou Peninsula in tropical South China, where a humid hot climate prevails because of the influence of continental and maritime monsoons as well as typhoons. Thus, microbial biofilms thrive on the stone monuments due to the preferable temperature and humidity over the year (Wang et al., 2021). Our previous work showed that most of them suffer from severe biodeterioration caused by diverse microorganisms, including fungi, cyanobacteria, and sulfur-oxidizing bacteria (Wang and Liu, 2021; Wang et al., 2021), resulting in a considerable loss of appearance (Figure 1). However, our further work indicated that micro-environments significantly affect community structures and biodeterioration processes of epilithic biofilms on the monuments (Meng et al., 2023). Contrary to our initial hypothesis of congruence between the biodeterioration of the monuments in the same yard, we observed divergent physicochemical features of the soils at the bottom of the monuments, highlighting the need for a nuanced framework capturing the keystone microorganism-metabolite interplay. Therefore, identifying the keystone taxa and metabolisms involved in microbial biodeterioration of the monuments is essential to bridge the link between microbial biofilm and biodeterioration in cultural heritage microbiology research (Luo and Gu, 2025).

Here, we used an integrative high-throughput sequencing and comparative metabolomic approach to unravel the biodeterioration of the Leizhou Stone Dog monuments. To avoid the divergence of abiotic deterioration, we selected the monuments in the same yard as the subject. We tracked the divergent physicochemical features of the soils at the bottom of the monuments and systematically compared the divergence and similarity of the microbial composition of the biofilms colonizing the monuments to identify the keystone taxa governing the biodeterioration of each stone monument. Furthermore, the correlation between the keystone taxa and physicochemical properties was evaluated to check the consistency of the observations of the species-based metabolisms of the keystone taxa, leading to the biodeterioration of the monuments.

## 2 Materials and methods

### 2.1 Sample collection

Samples were collected in January 2024 in Leizhou (110°5′57″E, 20°54′56″N), Guangdong Province, China. The local climatic conditions during the period were featured as follow: a typical monsoon prevailing, an average temperature of 23°C, and an average precipitation of 1,000 mm (relative humidity of about 70%), but no precipitation in 1 week prior to sampling. Five sets of stone dog sculptures suffering from deterioration in an open-air courtyard were selected and coded M0137, M0211, M0252, M0295, and M0307, respectively (Figure 1). Both the biofilm samples and soil samples were collected for each sculpture. The epilithic biofilms colonizing each sculpture were sampled in 5 cm × 5 cm squares via the non-destructive method. Meanwhile, about 10 g of the soils at the bottom of the sculpture were collected for chemical assays. All the samples were packaged and stored at 4°C before laboratory-based analyses.

### 2.2 Ion chromatography analysis

The base soil was thoroughly mixed before ion chromatography analysis (Dick and Tabatabai, 1979). Briefly, 1 g of the soil sample was added to 5 mL of distilled water. After 15 min of mixing on the vortex followed by 5 min centrifugation at 1,000*g*, the supernatant was filtered using the microporous membrane (0.22 μm in diameter) for subsequent determination of anion and cation concentrations. Upon the detection limit of ion chromatography, the initial concentration would be diluted if necessary. Then, the prepared supernatant was injected into an ion chromatograph to detect anion and cation concentrations. The DionexTM AquionTM ICS 2100 (Thermo Fisher Scientific, MA, United States) system determined the anion concentration with the column IonPac™ AS19 (4 × 250 mm). The cation concentration was examined using the Dionex™ ICS 6000 (Thermo Fisher Scientific, MA, United States) system, with the column IonPac™ CS12A (4 × 250 mm). The entire detection was carried out at 25°C.

### 2.3 DNA extraction and sequencing

Following the manufacturer’s introduction, the ALFA-SEQ Soil DNA Kit (50) (FINDROP, CN) was used to extract the genomic DNA of biofilm samples. The concentration and purity were detected by Thermo NanoDrop One (Thermo Fisher Scientific, MA, United States).

The V4-V5 regions of 16S rRNA genes were amplified using specific primers (Bacteria: 515F-GTGCCAGCMGCCGCGGTAA, 907R-CCGTCAATTCMTTTRAGTT; Archaea: 524F10extF-TGYCAGCCGCCGCGGTAA, Arch958RmodR-YCCGGCGTTGAV-TCCAATT), in which a 12-bp barcode was appended (Tamaki et al., 2011; Pires et al., 2012). Primers were synthesized by Invitrogen (Invitrogen, Carlsbad, CA, United States).

The length and concentration of the PCR products were detected by 1% agarose gel electrophoresis. Samples with bright main strips between 400–450 bp could be used for further assays. PCR products were mixed in equidensity ratios according to the GeneTools Analysis Software (Version 4.03.05.0, SynGene). Then, a mixture of PCR products was purified with E.Z.N.A. Gel Extraction Kit (Omega, USA).

Sequencing libraries were generated using NEBNext^®^ Ultra™ II DNA Library Prep Kit for Illumina^®^ (New England Biolabs, MA, United States) following the manufacturer’s recommendations, and index codes were added. The library quality was assessed on the Qubit@ 2.0 Fluorometer (Thermo Fisher Scientific, MA, United States). Finally, the library was sequenced on an Illumina Nova6000 platform, and 250 bp paired-end reads were generated (Guangdong Magigene Biotechnology Co., Ltd. Guangzhou, China). The high-throughput sequencing data supporting this study’s findings were available in the National Center for Biotechnology Information (NCBI) under accession no. PRJNA1170002.

### 2.4 Bioinformatics and taxonomy analyses

Downstream analysis of high-throughput sequencing data was carried out by QIIME2 (Quantitative Insights Into Microbial Ecology 2) (Bolyen et al., 2019). The primers were removed to obtain the paired-end Clean Reads using Cutadapt software (Martin, 2011), according to the primer information at the beginning and end of the sequence. The paired ends sequencing data were denoised and spliced, and chimeras were removed using the DADA2 algorithm (Callahan et al., 2016). The sequence quality was controlled by specifying the truncation length of the front and back ends. The quality statistics of the spliced sequences were carried out, and the lowest value was leveled. The operational taxonomic units (OTUs) were extracted at a threshold of 97% of identity among the reads. The OTUs were classified by the feature-classifier module in QIIME2 to obtain the taxonomy of bacteria and archaea, referring to the Sliva 16S rRNA (v.138.2).

### 2.5 Statistical analysis

One-way analysis of variance (ANOVA) was performed to assess the differences between chemical properties of the soils. Assuming the soils were mostly derived from underlying stone from biological activity, their chemical properties were then analyzed against microbial metabolism from biofilms using Waller-Duncan’s *post hoc* test in IBM SPSS v.25.0 (Chicago, IL, United States). Stacked columns were performed to display bacterial and archaeal community structures using the “ggplot2” package in R v.4.4.1.^2^ The heatmap showing the differences in genus abundance among different samples was standardized using the Z-Score method, and the calculation formula was as follows:


$$\text{value}=\frac{x-\mu }{\sigma }$$

where *μ* was the mean and *σ* was the standard deviation of the data (Singh and Singh, 2020).

Taxonomic trees of the shared OTUs of bacteria and archaea were generated by Cytoscape software with the node and edge files calculated by R according to the shared OTU table. To better explain the relationship between keystone taxa and biodeterioration, we employed Mantel’s test to compare different phenotypes with physicochemical properties of the base soils and identify the correlation of consistent biological information from functional results of metabolomic pathway analysis (Ten-Doméncech et al., 2021). Mantel’s test was carried out to examine the relationships between the chemical properties and the structure of the core taxa using the “linkET” and “ggplot2” packages (Huang, 2021). To mine the metabolism information of the microbial community, the algorithms PICRUSt2 and FAPROTAX were applied to predict the functional potential of the bacterial and archaeal community by 16S rRNA gene sequencing profiles. The PICRUSt2 predictions were mapped to the annotated genes catalog of the Kyoto Encyclopedia of Genes and Genomes (KEGG) database (Douglas et al., 2020). The FAPROTAX algorithm extrapolated taxonomic profiles of microbial communities into putative functional profiles based on a database of cultured microorganisms (Louca et al., 2016).

## 3 Results

### 3.1 Physicochemical properties of the soils under the bio-deteriorated monuments

**Figure 2:**
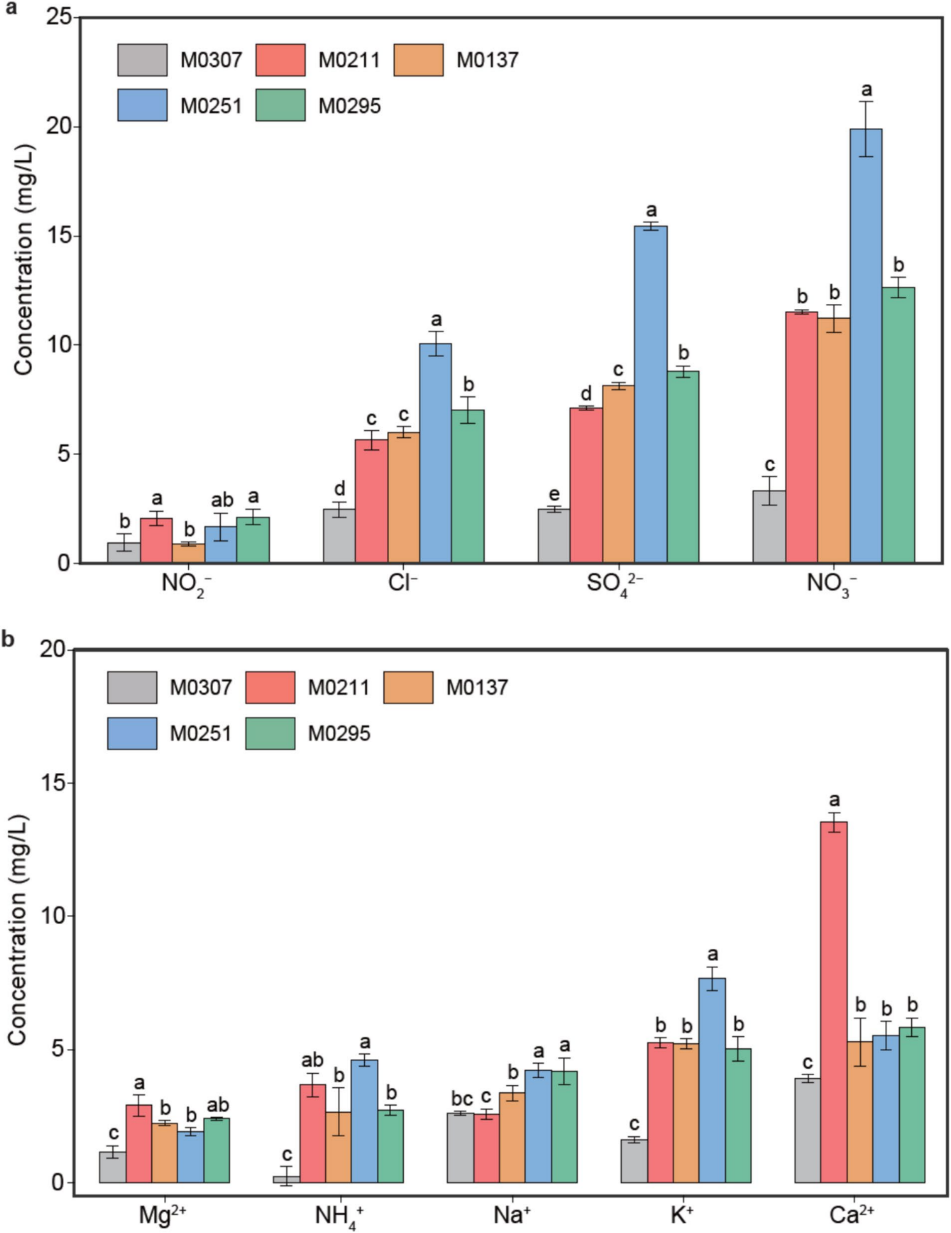
Physicochemical properties of the soils at the bottom of each sculpture. Contents of anions **(a)** and cations **(b)** in the soils.

Four anions (i.e., NO_2_^−^, Cl^−^, SO_4_^2−^ and NO_3_^−^) and five cations (i.e., Mg^2+^, NH_4_^+^, Na^+^, K^+,^ and Ca^2+^) were detected among the five samples (Figure 2). Generally, the contents of anions in each sample ranked as NO_3_^−^ > SO_4_^2−^ > Cl^−^ > NO_2_^−^, with NO_3_^−^ and SO_4_^2−^ being almost 5 and 4 times the content of NO_2_^−^, respectively (Figure 2a). Moreover, the contents of cations in each sample (except M0307) were ordered as Ca^2+^ > K^+^ > Na^+^ > NH_4_^+^ > Mg^2+^, with the contents of Ca^2+^ and K^+^ higher than the other three cations (*p* < 0.05, Figure 2b).

Despite the monuments located in the same yard, the content of each cation or anion exhibited significant differences among the samples (*p* < 0.05). For example, the contents of NO_3_^−^, SO_4_^2−^_,_ and Cl^−^ in M0251 and M0307 were the highest and the lowest among the samples, respectively. Similarly, the contents of cations in M0307 were the lowest compared with those in the other samples. Also, the content of Ca^2+^ in M0211 doubled that of Ca^2+^ in the other four samples, whereas K^+^, Na^+^, and NH_4_^+^ exhibited the highest content in M0251.

### 3.2 Diversity and composition of epilithic biofilms on the monuments

We investigated the biodiversity and composition of epilithic biofilms colonizing the monuments to unravel microbial communities relevant to the biodeterioration of the monuments (Wang and Liu, 2021; Meng et al., 2023). Generally, the bacterial diversity was ranked as M0295 > M0211 ≈ M0251 > M0307 ≈ M0137, whereas archaeal diversity was ordered as M0251 ≈ M0137 > M0295 ≈ M0307 > M0211 (*p* < 0.05, Supplementary Figure S1).

**Figure 3:**
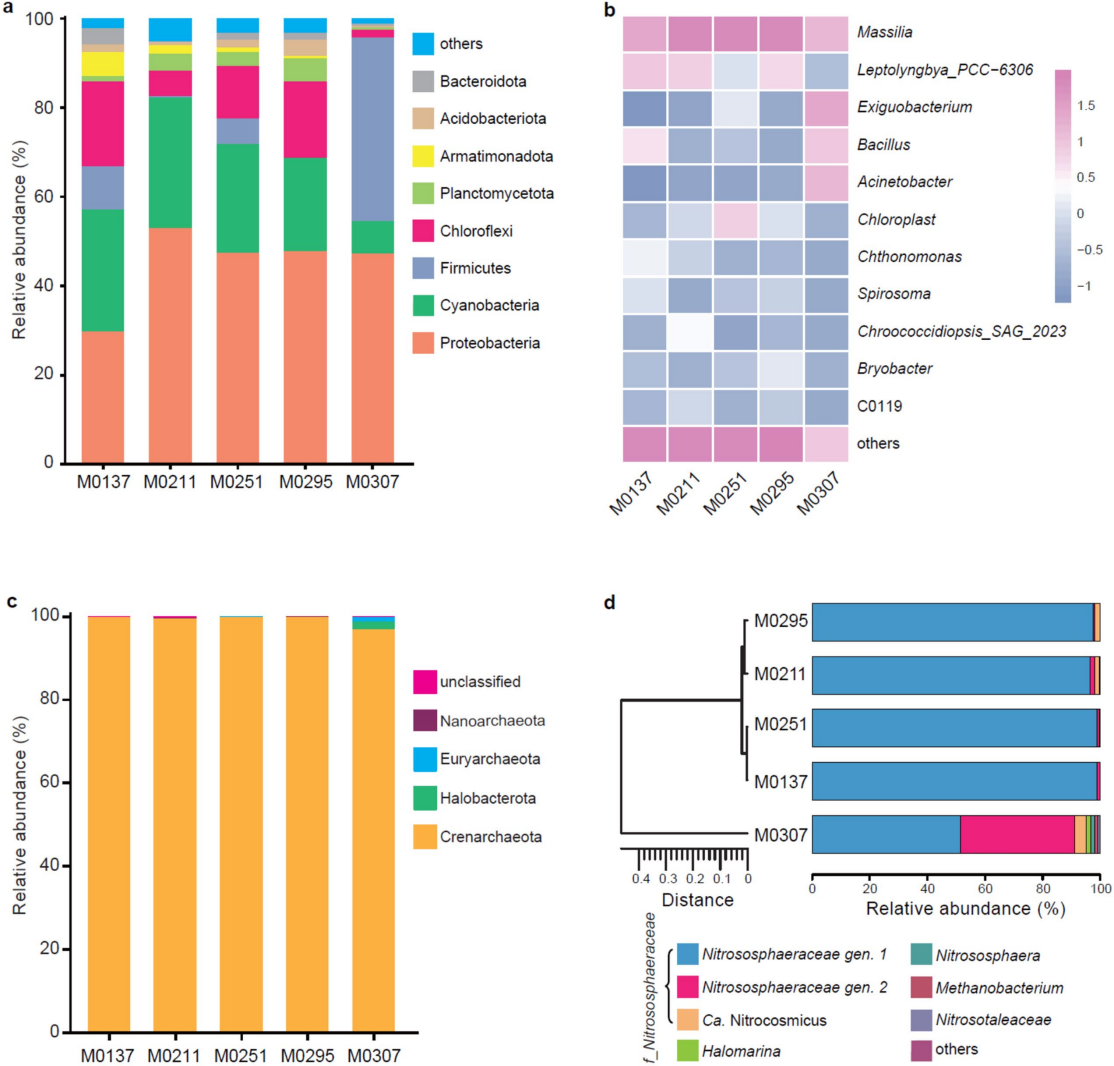
Bacterial and archaeal compositions of the epilithic biofilms colonzing the sculptures. **(a)** Bacterial populations at the phylum level. Others represent the phyla with the relative abundance less than 1%. **(b)** Bacterial populations at the genus level. Others include the unclassified genera and the genera with the relative abundance less than 1%. **(c)** Archaeal populations at the phylum level. **(d)** Composition of the archaeal genera. Others include the unclassified genera or the genera with the relative abundance less than 1%. On the left is a cluster tree drawn by similarity clustering based on different sample distance matrices.

Bacteria in all the biofilms mainly included eight phyla, such as Proteobacteria, Cyanobacteria, Firmicutes, and Chloroflexi (Figure 3a). Consistently, Proteobacteria were the predominant community, followed by two phototrophic phyla, Cyanobacteria and Chloroflexi, accounting for 35–50% of bacterial populations in the biofilms. Differentially, Proteobacteria and Firmicutes co-predominated and accounted for more than 90% of bacterial communities on the monument M0307, which might be a consequence of the distinctive physicochemical properties of the soil under M0307. Moreover, the most abundant genera listed in order were *Massilia*, *Leptolyngbya_PCC−6,306*, *Exiguobacterium*, *Bacillus*, *Acinetobacter*, *Chloroplast*, *Chthonomonas*, *Spirosoma*, *Chroococcidiopsis_SAG_2023*, *Bryobacter* and *C0119* (Figure 3b). Commonly, *Massilia* spp. were the predominant bacteria in all the biofilms. Differently, *Exiguobacterium*, *Bacillus,* and *Acinetobacter* predominated in bacterial communities on the monument M0307, *Leptolyngbya_PCC−6,306* predominated on M0137, M0211 and M0295, *Chloroplast* on M0251 and *Bacillus* on M0137.

Archaeal communities in the biofilms mainly included four phyla Crenarchaeota, Halobacterota, Nanoarchaeota, and Euryarchaeota, with Crenarchaeota accounting for more than 95% of the populations (Figure 3c). Interestingly, abundances of Halobacterota (~3%) and Euryarchaeota (~2%) in the biofilm on M0307 were significantly higher than the others. Moreover, archaeal populations were mainly affiliated to seven genera, including two unclassified genera of *Nitrososphaeraceae*, *Ca.* Nitrocosmicus, *Halomarina*, *Nitrososphaera*, *Methanobacterium* and *Nitrosotaleaceae* (Figure 3d). Notably, archaeal populations of M0295, M0211, M0251, and M0137 were purely predominated by an unclassified genus of *Nitrososphaeraceae*, which shared 90% of archaeal populations of M0307 with the other unclassified genus of *Nitrososphaeraceae*. Besides, *Ca.* Nitrocosmicus accounted for 4% of archaeal members of M0307 and 2% of archaeal members of M0295 and M0211. Members in the family *Nitrososphaeraceae* are typical ammonia-oxidizing archaea that convert ammonia to nitrite (Bei et al., 2024), while the latter could be further oxidized to nitrate by aerobic nitrite-oxidizing bacteria (e.g., *Bacillus* spp.) (Abeliovich, 2006; Daims et al., 2016). Thus, the predominance of the families *Nitrososphaeraceae* and *Bacillus* explains the observation that the content of nitrate is significantly higher than that of nitrite and/or ammonia in all the soils.

Although we had an overview of the microbial compositions of the epilithic biofilms on the bio-deteriorated monuments, the core taxa involved in the biodeterioration processes were still unclear. Thus, further exploration of the populations of the epilithic biofilms colonizing the monuments is required to unravel the keystone taxa that might lead to biodeterioration.

### 3.3 Keystone taxa of the epilithic biofilms

**Figure 4:**
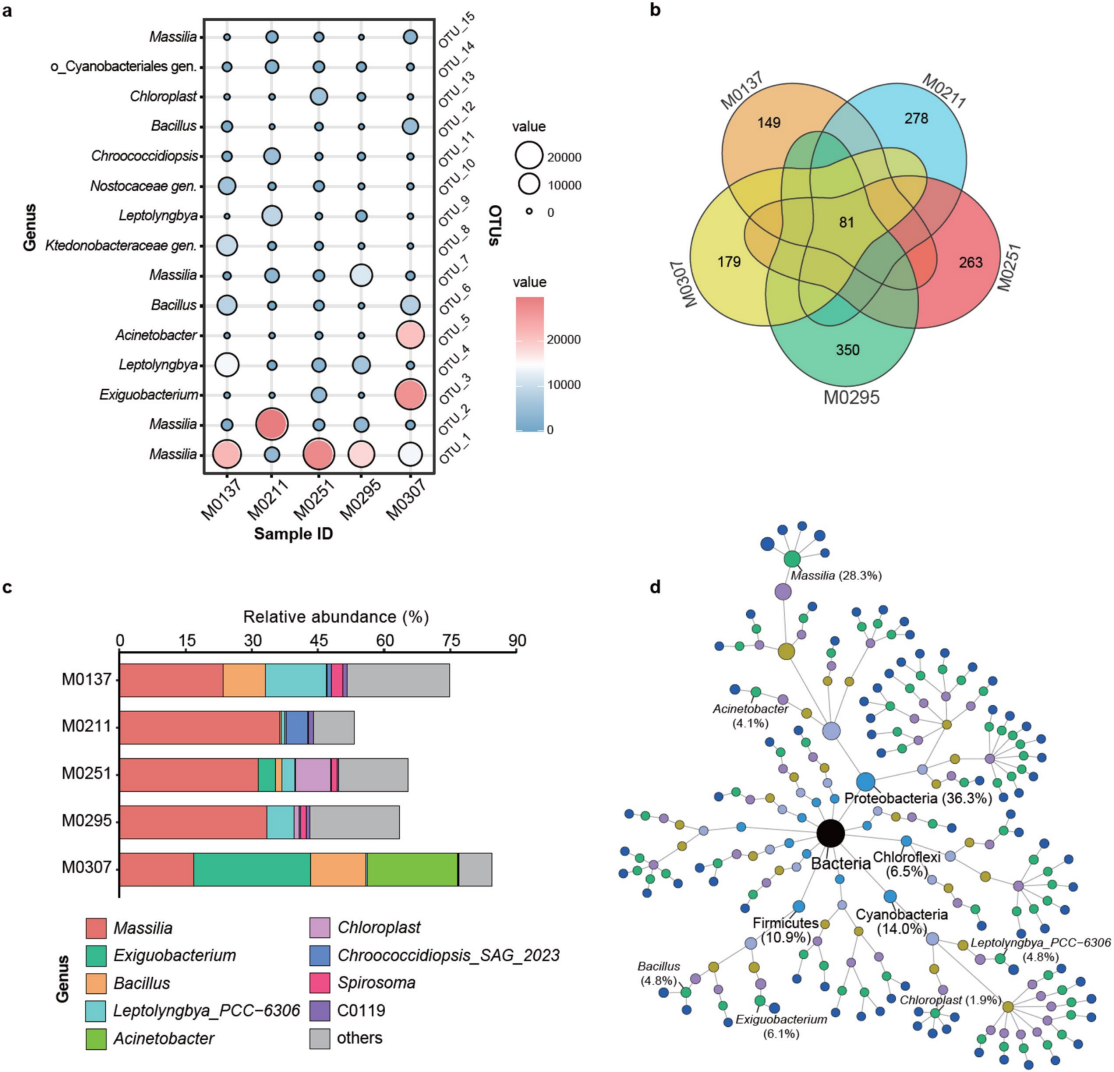
Distribution of the keystone bacteria in the epilithic biofilms. **(a)** Distribution of the keystone species across the biofilms. Bubble color and size represent the abundance distribution of each species. **(b)** Venn analysis of common and private bacterial populations among the biofilms. **(c)** Relative abundance distribution of the shared 81 species across the biofilms. Others indicate the unclassified members. **(d)** Taxonomic trees of the shared species. Color of the nodes represents the different taxonomic levels of domain, phylum, class, order, family, genus, and species. Size of the nodes reflects the relative abundance.

To figure out the keystone taxa relevant to the biodeterioration of the monuments, we further extracted and compared the predominant species across the biofilm communities. The distribution of the 15 most abundant OTU showed that members of the genera *Massilia*, *Exiguobacterium*, *Leptolyngbya*, *Acinetobacter*, *Bacillus*, *Chroococcidiopsis* and *Chloroplast* were the predominant bacterial species of all the biofilm communities (Figure 4a). Specifically, the predominant species in the biofilm M0137 were mainly affiliated to the genera *Massilia*, *Leptolyngbya*, *Bacillus*, and two unclassified genera of the families *Ktedonobacteraceae* and *Nostocaceae.* Bacterial communities of the biofilms M0211, M0251, and M0295 were mainly predominated by *Massilia* spp., followed by *Leptolyngbya* spp., *Chloroplast* spp., and *Leptolyngbya* spp., corresponding to the biofilm. Unlike the other biofilms, bacterial species in the biofilm M0307 mainly belonged to the two genera *Exiguobacterium* and *Acinetobacter*, followed by *Massilia* and *Bacillus*. The observations of the most abundant species were generally consistent with the composition of bacterial genera, which further confirmed the potential keystone taxa of these genera involved in the biodeterioration of the monuments.

Furthermore, we extracted the shared species across the bacterial communities (Figure 4b). As a result, it showed that the five biofilm samples shared only 81 species with most of the species owned by individuals, especially M0295, M0211, and M0251, suggesting the differentiation of bacterial communities among the biofilm samples. To identify which shared 81 species represented each sample, we investigated their abundance distribution among the biofilm samples. We found that these shared species mainly belonged to the genera *Massilia*, *Exiguobacterium*, *Bacillus*, *Leptolyngbya*, *Acinetobacter*, *Chloroplast*, *Chroococcidiopsis*, *Spirosoma* and *C0119* (Figure 4c). Together with the unassigned members, they represented the most bacterial populations of each sample, especially M0307 and M0137, indicating their possibility of keystone taxa. Specifically, the shared *Massilia* spp. represented more than 30% of bacterial populations of M0295, M0211, and M0251, which was consistent with the distribution of the most abundant species (Figure 4a). Besides the unassigned members, the shared *Massilia* spp., *Leptolyngbya* spp., and *Bacillus* spp. represented 20, 10, and 5% of the populations of M0137, respectively. In particular, 75% of the populations of M0307 were represented by the shared *Exiguobacterium* spp. (25%), *Acinetobacter* spp. (20%), *Massilia* spp. (18%) and *Bacillus* spp. (12%). These results outlined the keystone taxa of the bacterial populations in each sample. Moreover, the taxonomic tree of the shared 81 OTUs provided an overview of their affiliation and abundances in all the biofilm samples (Figure 4d). Based on the taxonomic tree, the shared *Massilia* spp. represented 28.3% of the total bacterial populations, followed by *Exiguobacterium* (6.1%), *Leptolyngbya* (4.8%), *Bacillus* (4.8%), *Acinetobacter* (4.1%), and *Chloroplast* (1.9%), which confirmed that they were the keystone taxa involved in the biodeterioration of the monuments.

**Figure 5:**
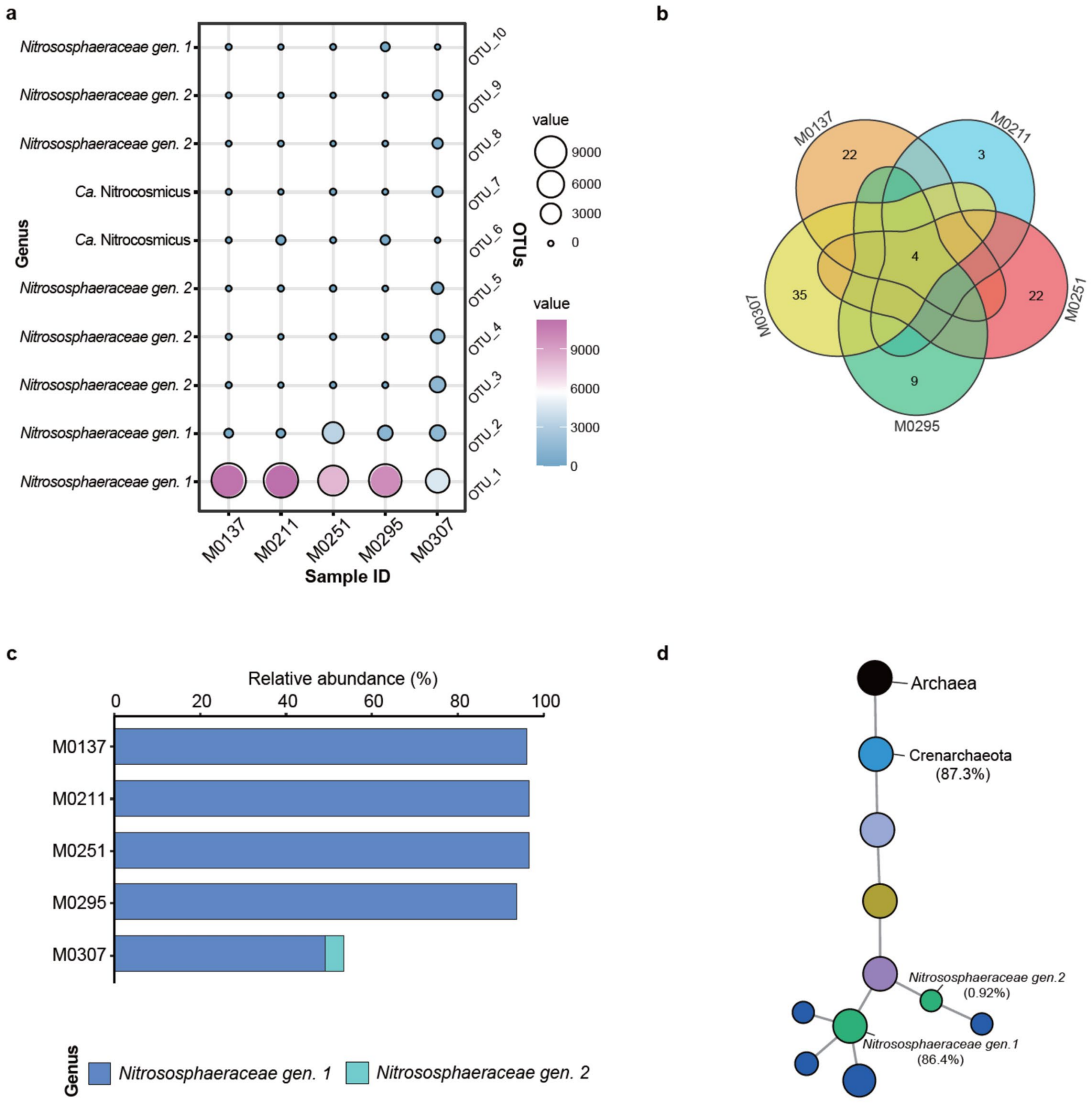
Distribution of the keystone archaea in the epilithic biofilms. **(a)** Distribution of the most abundant 10 archaeal species across the biofilms. Bubble color and size represent the abundance distribution of each species. **(b)** Venn analysis of common and private archaeal populations among the biofilms. **(c)** Relative abundance distribution of the shared species across the biofilms. **(d)** Taxonomic trees of the shared species. Color of the nodes represents the different taxonomic levels of domain, phylum, class, order, family, genus, and species. Size of the nodes reflects the relative abundance.

By contrast, the archaeal populations were relatively simple. The most abundant 10 OTUs indicated that they were affiliated to the two genera of the family *Nitrososphaeraceae* (Figure 5a), especially the *Nitrososphaeraceae gen. 1*, suggesting their keystone taxa of the archaeal populations. The archaeal species (OTU-1) predominated the archaeal populations of all the biofilms, especially M0137, M0211, and M0295. Another archaeal species (OTU-2) was the second predominant member of the archaeal populations of M0251. Significantly, the archaeal populations of M0307 were not only predominated by the species (OTU-1) but also by other four species (i.e., OTU-2, OTU-3, OTU-4, and OTU-5), indicating the differentiation of M0307 to the other four biofilms. Moreover, the number of the shared species was four, with M0307 owning the unique species (Figure 5b), further suggesting its differentiation in the archaeal community. Unexpectedly, the common species were all affiliated to *Nitrososphaeraceae gen. 1* and represented more than 90% of the archaeal populations of the biofilm samples, except M0307 (Figure 5c). In M0307, the archaeal populations were represented by *Nitrososphaeraceae gen. 1* (48%) and *Nitrososphaeraceae gen. 1* (4%). Further, the taxonomic tree of the shared archaeal OTUs confirmed *Nitrososphaeraceae gen.1* as the keystone taxa in all the biofilm samples (Figure 5d).

### 3.4 Correlations of keystone taxa with biodeterioration

**Figure 6:**
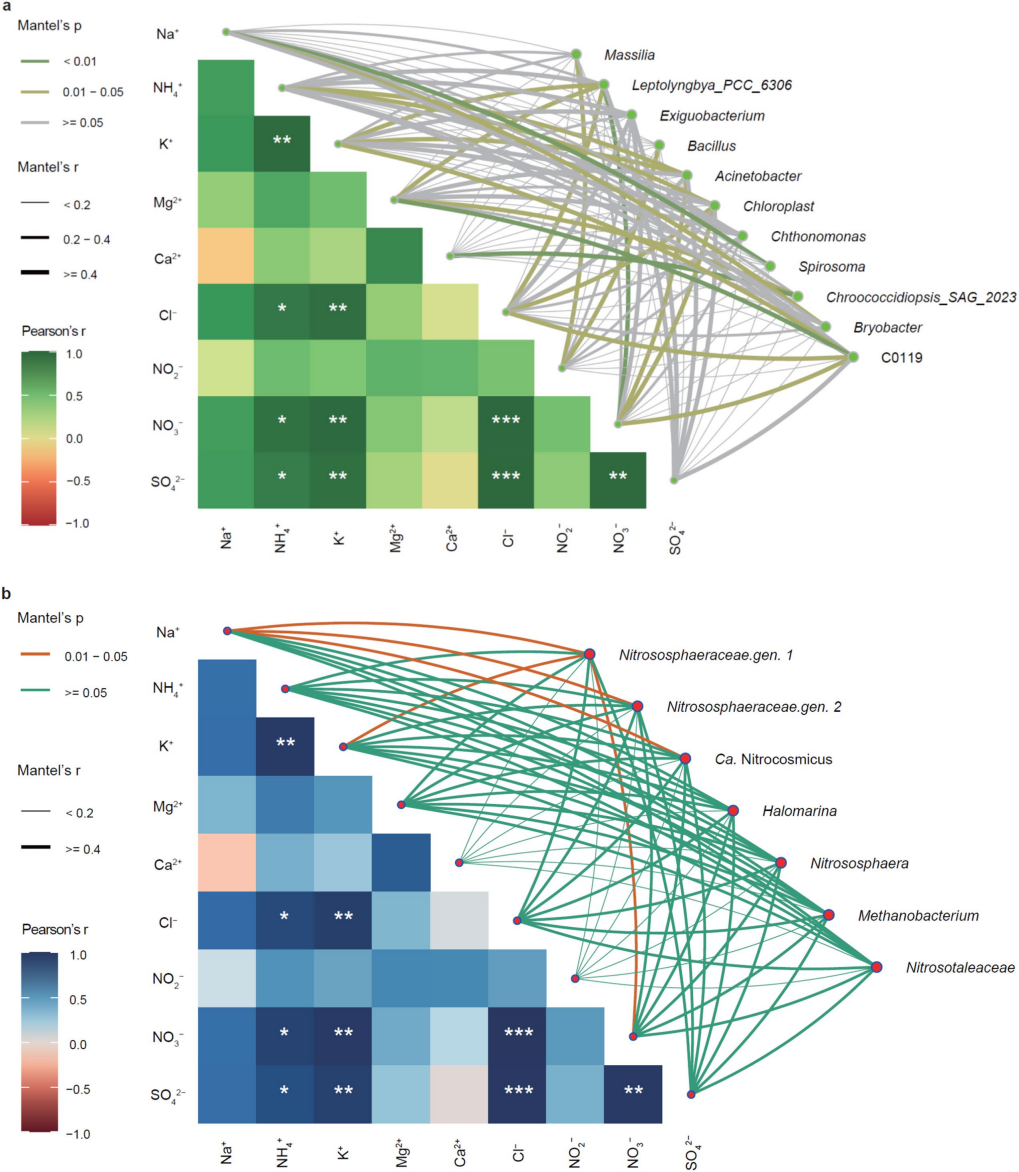
Correlation networks between keystone taxa and physicochemical properties. **(a)** Correlations between keystone bacteria and physicochemical properties. **(b)** Correlations between keystone archaea and physicochemical properties. Pearson’s coefficient indicates the correlation between different physicochemical properties, whereas the correlation of Mantel’s test shows the effect of physicochemical properties on the keystone bacterial and archaeal communities. Line thickness indicates the degree of correlation and *p*-value represents the degrees of significance, *p < 0.05, **p < 0.01, and ***p < 0.001.

The Mantel’s test showed that the contents of Cl^−^, SO_4_^2−^ and NO_3_^−^ were significantly correlated with the contents of NH_4_^+^ (*p* < 0.05) and K^+^ (*p* < 0.01), instead of Ca^2+^ or Mg^2+^ (Figure 6). Moreover, the content of Cl^−^ was extremely significantly correlated with the contents of SO_4_^2−^ and NO_3_^−^ (*p* < 0.001), while the content of K^+^ was significantly correlated with that of NH_4_^+^ (*p* < 0.01).

In the bacterial keystone taxa (Figure 6a), the genera *Spirosoma*, *Chroococcidiopsis*, and *C0119* were highly significantly correlated with Na^+^, Ca^2+^_,_ and Mg^2+^ (*p* < 0.01), respectively, indicating they might be the keystone taxa leading to mineral solutions of stone monuments (Keshari and Adhikary, 2014; Yu et al., 2024). Importantly, the two genera *Massilia* and *Bacillus* were solely significantly correlated with NO_2_^−^, whereas NO_3_^−^ was significantly correlated with *Leptolyngbya*, *Acinetobacter*, *Chloroplast*, and *C0119*, with *Acinetobacter* and *Chloroplast* correlated with NH_4_^+^ as well, suggesting they were potential keystone bacteria involved in nitrification and/or denitrification (Qiao et al., 2020). Moreover, the most abundant *Nitrososphaeraceae gen 1* was significantly correlated with NO_3_^−^, but we found no potential keystone taxa correlated with SO_4_^2−^ (Figure 6b).

### 3.5 Potential metabolic processes involved in biodeterioration

**Figure 7:**
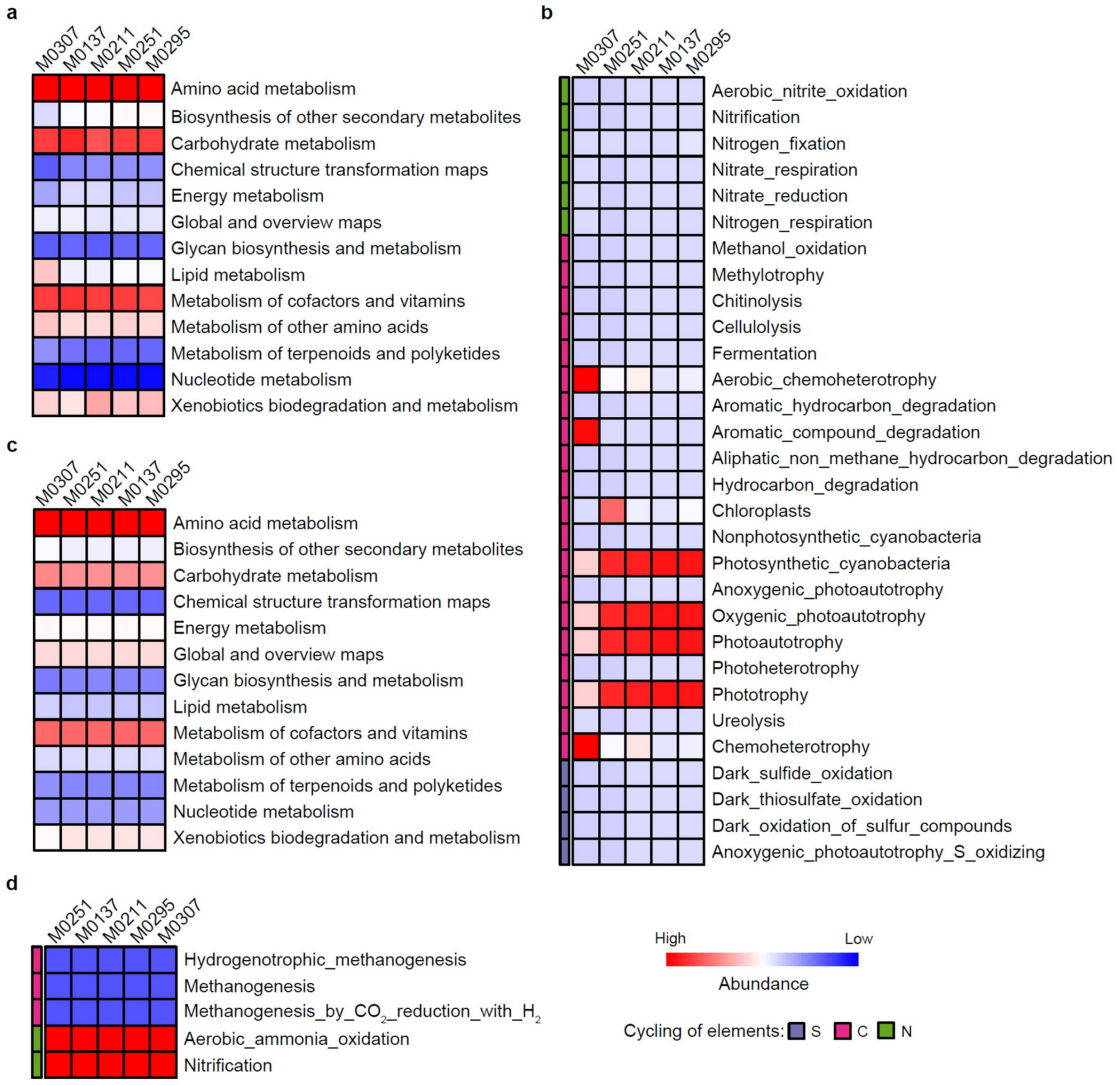
Keystone metabolisms of the epilithic biofilms. **(a)** General metabolisms of bacterial populations of the epilithic biofilms. **(b)** Bacterial metabolic processes involved in biogeochemical cycling of C, N, and S. **(c)** General metabolisms of archaeal populations of the epilithic biofilms. **(d)** Archaeal metabolic processes involved in biogeochemical cycling of C, N, and S. The relative abundance of the general metabolisms was predicted by the PICRUSt2 analysis, whereas that of the metabolic processes responsible for biogeochemical cycling of C, N, and S was predicted by the FAPROTAX analysis.

Generally, there were no significant differences in the total metabolisms in bacterial populations among the samples (Figure 7a), where amino acid metabolism, carbohydrate metabolism, and metabolisms of cofactors and vitamins were the predominant processes. Specifically, the differences were mainly found in C metabolisms rather than N and S metabolisms (Figure 7b). Bacterial populations of the biofilm M0307 were characterized by chemoheterotrophy and aromatic compound degradation. In contrast, bacterial populations of the other four biofilms consistently relied on phototrophic processes, corresponding to the dominant phototrophic taxa in epilithic biofilms (Méndez et al., 2024), such as *Leptolyngbya* and *Chroococcidiopsis*.

Similarly, the general metabolisms of archaeal populations among the samples also exhibited no differences, with amino acid metabolism, carbohydrate metabolism, and metabolisms of cofactors and vitamins predominated over the general metabolisms (Figure 7c). Interestingly, aerobic ammonia oxidation and nitrification were consistent characteristics of archaeal populations of all the samples (Figure 7d), which further confirmed our observations of the keystone taxa of the family *Nitrososphaeraceae* (Figure 6b).

## 4 Discussion

We used an integrative high-throughput sequencing and comparative metabolomic approach to unravel the biodeterioration of the Leizhou Stone Dog monuments. The divergence and similarity of the microbial composition of the biofilms colonizing the monuments were compared to identify the keystone taxa governing the biodeterioration of each stone monument. The correlation between the keystone taxa and physicochemical properties confirmed the consistency of the observations. In addition, the species-based functional information of the keystone taxa supported the potential metabolisms leading to the biodeterioration of the monuments.

We examined the contents of soluble salts in the soils deposited at the bottom of the bio-deteriorated monuments to track the potential chemical processes involved in biodeterioration. Collectively, the observations indicate that abundant nitrates and sulfates are accumulated in the soils. The Mantel’s test showed that excessive sulfuric and nitric acids might be needed to maintain the residual Ca^2+^ or Mg^2+^ in solution in the soils. Meanwhile, the significantly higher contents of SO_4_^2−^ and NO_3_^−^ than those of Ca^2+^ and Mg^2+^ could also support this inference (Figure 2). These observations suggest significant differences in the contents of soluble minerals in the soils, indicating that different intensities of deterioration occur on the monuments. Such deterioration could be attributed to both abiotic and biotic processes depending on the environment the monuments were exposed to (Liu et al., 2022b). The monuments were all in the same yard and suffered the same abiotic impacts (Figure 1). Thus, biodeterioration caused by epilithic biofilms on the monuments would be the most likely reason to explain the differences in these observations.

While physicochemical features elucidated the potential deterioration processes, the divergence of microbial communities highlighted the contributions of biotic deterioration rather than abiotic deterioration because the monuments were exposed to the same environment. Notably, the community compositions across the biofilm samples indicated common and divergent populations for each sample, consistent with our previous observation that the micro-environment determines community structures and biodeterioration processes of epilithic biofilms (Meng et al., 2023). For example, the most abundant *Nitrososphaeraceae gen 1* was significantly correlated with NO_3_^−^ (Figure 6b), confirming that they were the keystone taxa responsible for nitrification that have been reported to be responsible for biodeterioration of stone monuments (Meng et al., 2016; Ding et al., 2021). Consistently, the two genera of *Nitrososphaeraceae* and *Ca.* Nitrocosmicus were significantly relevant to Na^+^ and K^+^, suggesting they are the keystone taxa responsible for the salt efflorescences of the monuments (Mihajlovski et al., 2019). This highlights the importance of the micro-environment in shaping the biofilm community colonizing stone monuments and their biodeterioration, suggesting the necessity of a case-by-case treatment before any solution is used to protect from microbial attacks (Liu et al., 2022a).

**Figure 8:**
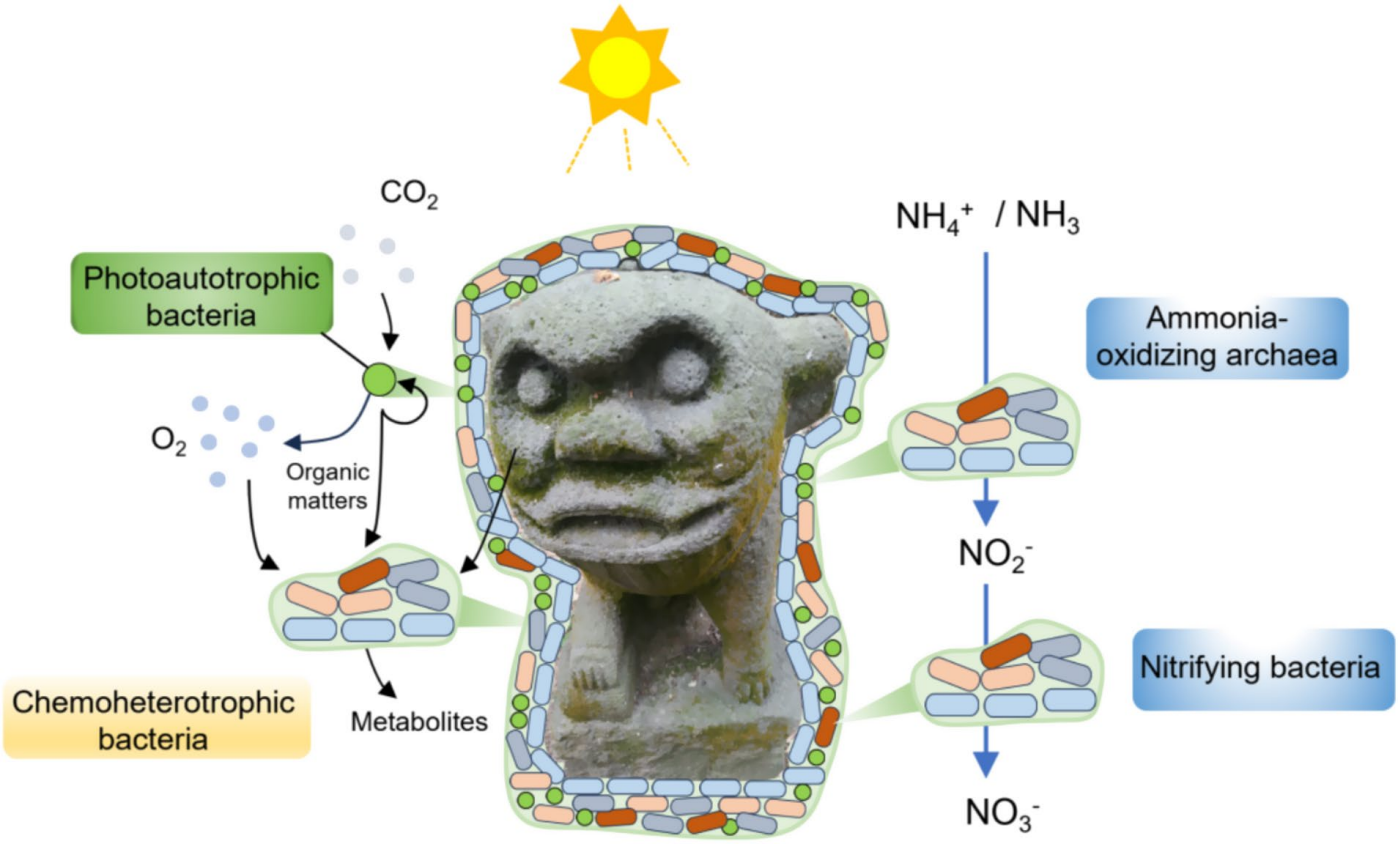
Keystone taxa and metabolisms of epilithic biofilms involved in the biodeterioration of the Leizhou Stone Dog monuments.

We also predicted the potential metabolisms of the keystone taxa to further identify the C, N, and S metabolic pathways that might be relevant to the biodeterioration. Collectively, the observations of the potential metabolisms were consistent with the keystone taxa in the biofilms colonizing the deteriorated monuments. Thus, we claimed that photoautotrophy and/or chemoheterotrophy of bacterial populations and nitrification of archaeal populations would be the keystone metabolic processes leading to the biodeterioration of the Leizhou Stone Dog monuments (Figure 8). Future work aims to elucidate causal mechanisms through complementary methods such as mapping multi-omics data into metabolic pathways using chromatographic techniques, stable notable labeling, and microbial isolation studies, especially the keystone taxa. We acknowledge the limitations of relying solely on direct species-based functional prediction for metabolisms involved in biodeterioration, including the inability to differentiate active microbes or determine metabolites, as well as issues with sampling errors. Moving forward, implementing this culture-independent technique alongside culture-dependent and chemical techniques could provide a better understanding of the complex biodeterioration processes of stone monuments (Liu et al., 2022a).

Furthermore, incorporating longer timescales and expanded climate representation will further refine our understanding of the succession of the most relevant keystone taxa and metabolisms involved in the biodeterioration of the stone monuments. For example, continuous *in-situ* detection and comparison of the incident radiation on the monument surfaces between different seasons could greatly benefit a full explanation why the abundance or photosynthetic yields of photoautotrophic microorganisms evolves in the biofilms (Figueroa et al., 2017). Moreover, we observed some cyanobacterial inhabitants of low light (Hauer et al., 2015), such as *Leptolyngbya* and *Chroococcidiopsis*, which were also detected on other cultural heritage stone buildings in tropical and subtropical climates (Keshari and Adhikary, 2014; Ortega-Morales et al., 2019), indicating that a upper-to-lower layer analysis of microbial composition is required to confirm the divergence of spatial distribution in the epilithic biofilms (Czerwik-Marcinkowska and Massalski, 2018). Interestingly, despite having some abundant genera in common with other observations (Silva et al., 2022), such as *Spirosoma* and *Massilia*, their contribution to biodeterioration of stone monuments is still unclear. However, the genus *Massilia* are typical soil bacteria of the *Oxalobacteraceae*, but their members are often pigmented bacteria (Yang et al., 2019), indicating their involving in discoloration of stone monuments. By integrating keystone taxa and metabolism formulas into future mechanistic models of biodeterioration diagnosis and therapy, we can gain a focused picture of measures for the sustainable conservation of stone monuments from microbial damage.

In conclusion, we have successfully identified the keystone taxa and metabolisms involved in microbial biodeterioration of the Leizhou Stone Dog monuments. We propose an integrative culture-independent and culture-dependent approach to the necessity of a case-by-case diagnosis of the keystone taxa and metabolisms before any therapy, shedding light on the complex biodeterioration processes of stone monuments and advancing the conservation science of cultural heritage.

## Data Availability

The original contributions presented in the study are publicly available. This data can be found at: https://www.ncbi.nlm.nih.gov/, accession number: PRJNA1170002.

## Footnotes

^1^ https://whc.unesco.org/en/list/

^2^ https://www.r-project.org/
