# Supplementary material for: Identifying keystone taxa and metabolisms of epilithic biofilms is crucial to the conservation of stone heritage from biodeterioration
Source: Front Microbiol. 2025 May 27;16:1600865. doi: 10.3389/fmicb.2025.1600865 (PMC12150296; doi:10.3389/fmicb.2025.1600865)
Supplement: Supplementary file 1 [file Data_Sheet_1.PDF]

Supplementary information

Identifying keystone taxa and metabolisms of epilithic biofilms is crucial to the conservation of stone heritage from biodeterioration

Chenchen Ma[1, †], Xiaoying Zhang[1, †], Fasi Wu[2] & Xiaobo Liu[1, *]

[1] School of Environmental and Biological Engineering, Nanjing University of Science and Technology, 200 Xiaolingwei Street, Nanjing, Jiangsu 210094, China.

[2] National Research Center for Conservation of Ancient Wall Paintings and Earthen Sites, Department of Conservation Research, Dunhuang Academy, Dunhuang, Gansu 736200, China.

[†] These authors contributed equally: Chenchen Ma and Xiaoying Zhang.

[*] To whom the correspondence should be addressed:

Xiaobo Liu (xbliu@njust.edu.cn)

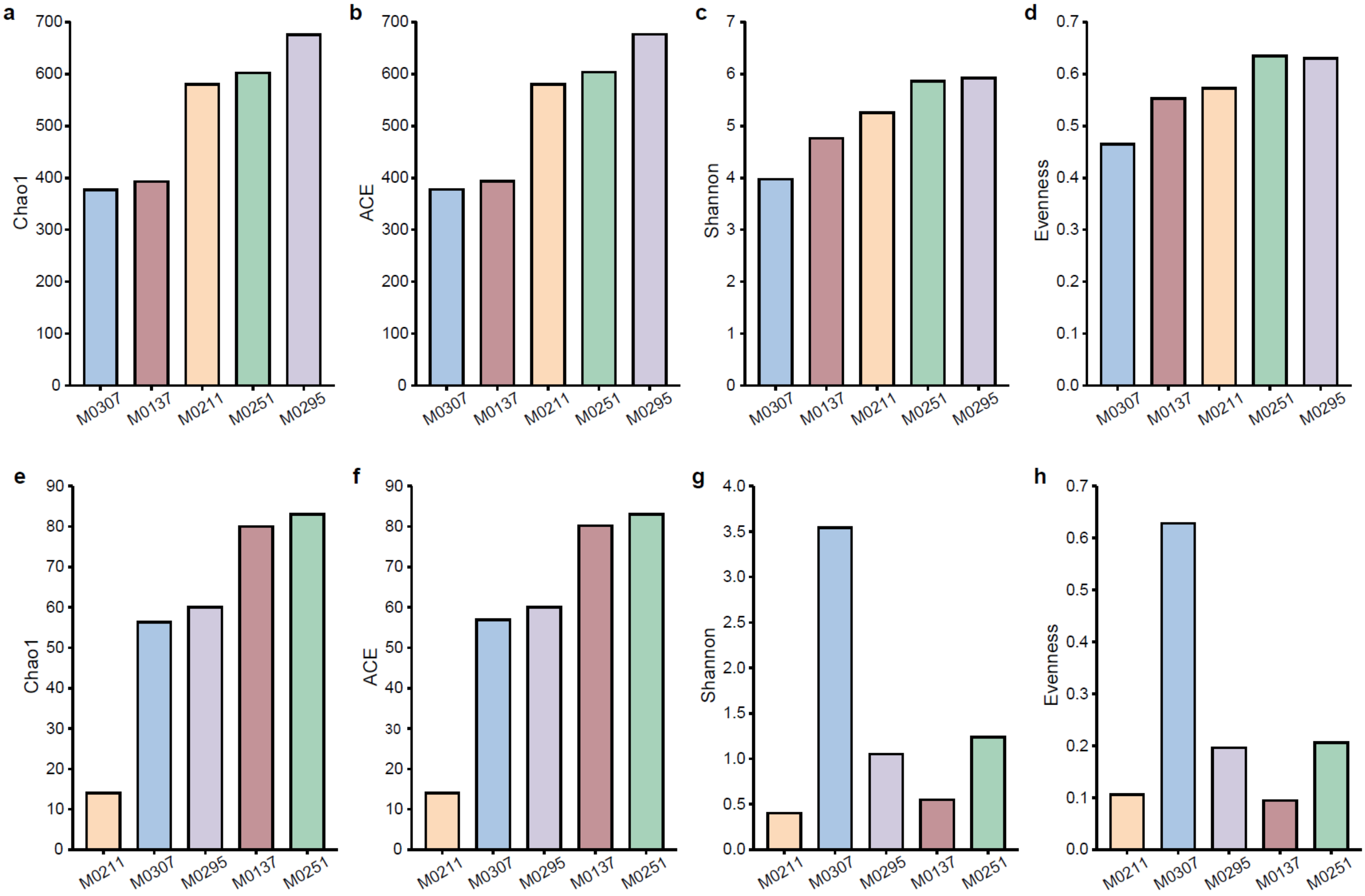

Fig. S1. Sample 16S bacterial (**a**-**d**) and archaeal (**e**-**h**) alpha diversity analysis. (**a**, **e**) Chao1 index; (**b**, **f**) ACE index; (**c**, **g**) Shannon index; (**d**, **h**) Evenness index.
